# Chalcogen Bonding in Co-Crystals: Activation through 1,4-Perfluorophenylene vs. 4,4′-Perfluorobiphenylene Cores

**DOI:** 10.3390/molecules26134050

**Published:** 2021-07-02

**Authors:** Arun Dhaka, Olivier Jeannin, Emmanuel Aubert, Enrique Espinosa, Marc Fourmigué

**Affiliations:** 1 CNRS, ISCR (Institut des Sciences Chimiques de Rennes) UMR 6226, Univ Rennes, 35000 Rennes, France ; arun.dhaka@univ-rennes1.fr (A.D.); olivier.jeannin@univ-rennes1.fr (O.J.); 2 CNRS, CRM2, Université de Lorraine, 54000 Nancy, France ; emmanuel.aubert@univ-lorraine.fr (E.A.) ; enrique.espinosa@univ-lorraine.fr (E.E.)

**Keywords:** chalcogen bonding, sigma-hole activation, crystal engineering, Lewis-base

## Abstract

The ability of alkylseleno/alkyltelluroacetylenes such as bis(selenomethylethynyl)-perfluorobenzene (**4F-Se**) to act as a ditopic chalcogen bond (ChB) donor in co-crystals with ditopic Lewis bases such as 4,4′-bipyridine is extended here to the octafluorobiphenylene analog, 4,4′-bis(selenomethylethynyl)-perfluorobiphenyl (**8F-Se**), with the more electron-rich 4,4′-bipyridylethane (**bpe**), showing in the 1:1 (**8F-Se**)•(**bpe**) co-crystal a shorter and more linear C−Se•••N ChB interaction than in (**4F-Se**)•(**bpe**), with Se•••N distances down to 2.958(2) Å at 150 K, i.e., a reduction ratio of 0.85 vs. the van der Waals contact distance.

## 1. Introduction

Crystal engineering strategies are manifested by the choice of intermolecular interactions owing to their strength, directionality and predictability that allow one to transfer the packing instructions in a molecule [1]. In this context, halogen bond (XB) [2,3], a subset of sigma-hole interactions, has emerged as an indispensable tool due to its significant linear directionality and tuneable sigma-hole activation that opens the door for a wide range of applications [4,5,6]. Similarly, chalcogen bond (ChB) [7] is an interaction between an electron-depleted site of an activated chalcogen atom and an electron-rich site of a Lewis-base [8,9]. Chalcogen bond, being a sister non-covalent interaction to halogen bond [10,11], has been mostly targeted to follow the trails of halogen bond in the field of crystal engineering but it is often limited by the presence of two sigma holes on chalcogen atoms. At variance with mono-valent halogen atoms, one extra valency of Se/Te can indeed invite additional changes in crystal packing that consequently dilutes the predictability of ChB interaction. Furthermore, organic selenides/tellurides are more reactive and are synthetically far less investigated compared to organic halides. Despite that, over the years, interesting ChB donors have been developed and some earlier examples include S(CN)_2_ [12], Se(CN)_2_ [13], 1,2,5-chalcogenadiazoles [14,15], 1,2-chalcogenazole N-oxides [16,17], or alkyltelluroalkynyl derivatives [18,19] that self-associate into discrete solid-state structures. To recover the linear directionality characteristic of halogen bonding interactions, chalcogen atoms are typically disymmetrized with only one strongly electron-withdrawing substituent to force a digger electron depletion along one favored single direction, as recently observed in organic selenocyanates, such as 1,4-*bis*(selenocyanatomethyl)-benzene that forms 1-D chains with 4,4′-bipyridine through short and directional Se•••N contacts [20,21,22,23]. The interaction strength parallels the degree of activation in both donor and acceptor molecules, an aspect often used in crystal engineering to quantify the robustness of a supramolecular motif.

**Scheme 1:**
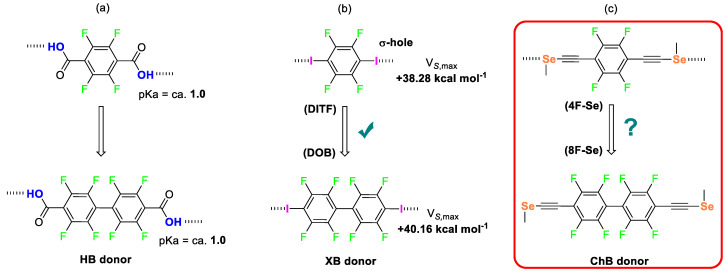
Activation through 1,4-perfluorophenylene vs. 4,4′-octaperfluorobiphenylene in different types of intermolecular interactions: (**a**) hydrogen bonding, (**b**) halogen bonding and (**c**) chalcogen bonding. See reference [28] for calculated V*_S_*,_max_ values for **DITF** and **DOB**.

In this regard, hydrogen and halogen bonding interactions have been extensively utilized to selectively tune the strength of intermolecular interactions in co-crystals by systematically treating either a donor with various acceptors of different Lewis-base character or an acceptor with various donors exhibiting different modes of activation. However, chalcogen bonding still remains less explored in this direction and one such example includes a recent study by Bryce et al. on investigations of ChB strength in a series of co-crystals between 3,4-dicyano-1,2,5-seleno/telluro-diazole acting as ChB donor and various N-oxide and pyridyl derivatives acting as ChB acceptors [24,25]. Rigid ChB donors such as alkylseleno/alkyltelluroacetylenes were recently demonstrated to exhibit a strong-sigma hole activation and remarkable control of directionality in co-crystal formation with bipyridyl derivatives, allowing for a fine tuning of Ch•••N interaction strength within the chalcogen-bonded 1-D chain motif [26]. In addition, a recent investigation of the U-shaped ChB donor 1,8-bis(telluromethylethynyl)-anthracene (BTMEA) with a series of (strong to weak) bipyridyl derivatives suggested that the strongest Te•••N interaction was indeed associated with the strongest Lewis-base used in this study [27]. Such fundamental studies become very crucial to understanding the limitations of chalcogen bond formed under different chemical and electronic environments. Surprisingly, the reverse situation where ChB strength can be tuned in a supramolecular motif through structural modification of the chalcogen bond donor itself is much less explored. In this context, an interesting example based on XB (Scheme 1b) showed that sigma-hole on iodine atom is rather enhanced when moving from 1,4-diiodotetrafluorobenzene (**DITF**) to 4,4′-diiodooctafluorobiphenyl (**DOB**) that incorporates a stronger activating core. This was also evident from V_S,max_ values of +38.28 and +40.16 kcal mol^−1^ (mapped on the electron density surface cut at the 0.002 e/Å^3^ level) observed for **DITF** and **DOB** respectively [28]. Additionally, their co-crystals with nicotine (**A1**), formed through halogen bonds, reveal that the strongest N•••I interaction was present in the structure of **DOB**•A1 with distance 2.808 Å (RR = 0.79) vs. 2.869 Å (RR = 0.81) found in **DITF**•A1 [28]. On contrary, a study by Aakeroy et al. on a series of co-crystals between these XB donors and heteroaryl-2-imidazoles (**A2**), driven through XB and HB, revealed that the strongest N•••I interaction was always associated with **DITF**•A2 structures in contrast to **DOB**•A2 co-crystals [29]. Hence, although the electrostatic surface potential suggests a stronger sigma-hole activation in **DOB** over **DITF**, this information is not explicitly reflected in their ability to form co-crystals with Lewis-base. Another element of comparison is brought by the pKa_1_ values of their analogous dicarboxylic acid derivatives (Scheme 1a) which were estimated to be approximately the same, indicating a negligible effect of the core on hydrogen bonding behavior [30].

Inspired by these results, we present herein a case study on the modulation of chalcogen bond with respect to the activation core in two ChB donors, namely 1,4-bis(selenomethylethynyl)-perfluorobenzene (**4F-Se**) vs. 4,4′-bis(selenomethylethynyl)-perfluorobiphenyl (**8F-Se**) (Scheme 1c).

## 2. Results and Discussions

**Figure 1:**
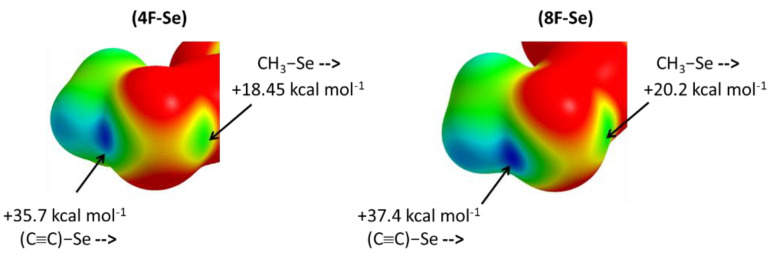
Electrostatic potential map of **BTMEA** in an optimized geometry showing the deep electron-depleted area (blue regions) in the prolongation of (C≡C)−Se bonds and a less significant one in the prolongation of the (CH_3_)−Se bonds. Color range from 0.0 (red) to 37.7kcal/mol (blue) and isosurfaces of electron density at ρ = 0.002 a.u.

The tendency of a molecule to engage in different types of intermolecular non-covalent interactions where electrostatics, besides dispersion, play a significant role can primarily be anticipated by mapping the electrostatic potential (ESP) on molecular surfaces. ESP calculations for our two ChB donors revealed the presence of a significant σ-hole on the Se atoms along the (C≡C)−Se bond with V*_S_*_,max_ values of +35.7 kcal mol^−1^ and +37.4 kcal mol^−1^ for donors **4F-Se** and **8F-Se** respectively (Figure 1). These values clearly indicate a comparatively larger activation of the σ-hole in **8F-Se**, demonstrating that the perfluorobiphenyl core plays an additional activating effect with respect to the perfluorophenyl one incorporated in **4F-Se.** A less significant second σ-hole is also present along the CH_3_−Se bonds in both **4F-Se** and **8F-Se** donors, with V*_S_*,_max_ values of respectively +18.45 kcal mol^−1^ and +20.2 kcal mol^−1^ establishing potentially a secondary directional preference of these donors toward molecular assembly.

**Scheme 2:**
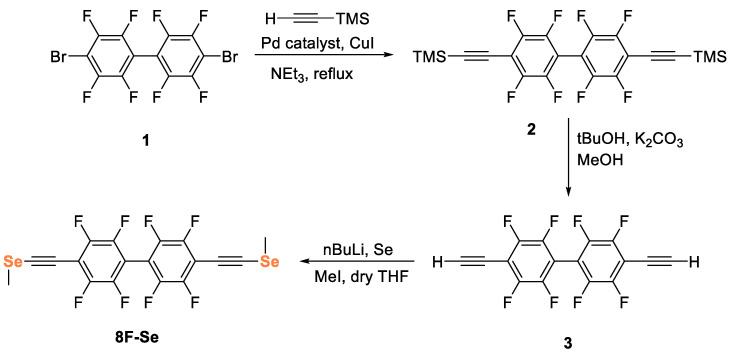
Synthesis route to the ChB donor **8F-Se**.

The synthesis of the new ChB donor **8F-Se** is outlined in Scheme 2 and starts with commercially available 4,4′-dibromoperfluorobiphenyle to first obtain compound **2** through Sonogashira coupling reaction. Subsequently, silyl deprotection followed by lithiation with Se metal and alkylation with MeI furnished the desired donor **8F-Se** in good yield. The same strategy starting with 1,4-diiodotetrafluorobenzene is then followed to obtain **4F-Se** as described in our previous report [26].

**Figure 2:**
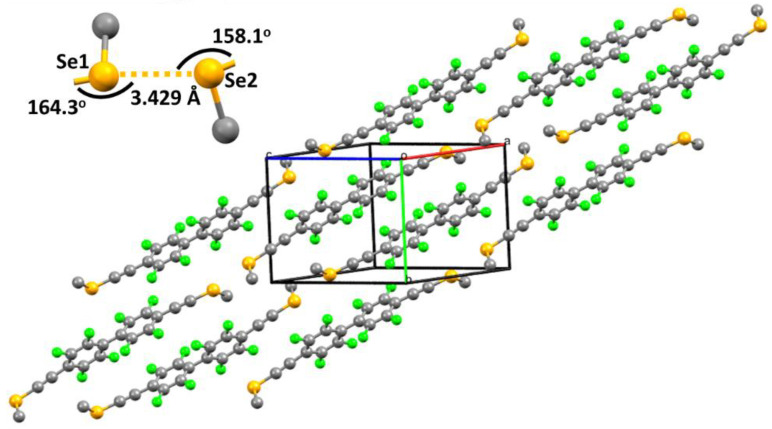
Crystal packing of **8F-Se** showing type-I Se•••Se interactions. Hydrogens are omitted for clarity.

Molecule **8F-Se** crystallizes in the triclinic system (SG $P\bar{1}$) into 1-D chains driven through Se•••Se contacts with the shortest intermolecular distance at 3.429 Å, which corresponds to a reduction ratio (RR) of 0.90 relative to the sum of van der Waals radii (2 × 1.90(Se) = 3.80 Å). The C−Se•••Se bond angle values of 164.3° and 158.1° suggest these Se•••Se interactions to be of type-I (Figure 2). The molecule is in general position with a torsion angle of 57.54° between both aromatic rings. Concerning the crystal structure of donor **4F-Se**, two different polymorphs in monoclinic (SG P2_1_/c) and tetragonal (SG I4_1_/a) systems were reported in our previous paper [26].

To further evaluate this difference in sigma-hole activation, we decided to co-crystallize both donors with various ditopic Lewis-bases to assess their ability to form chalcogen bonded 1D chains. We could obtain co-crystals from both ChB donors **4F-Se** and **8F-Se** with the 1,2-bis(4-pyridyl)ethane (**bpe**) as Lewis-base. Data were collected for both co-crystals at room temperature and at 150 K, in order to evaluate also the evolution of the ChB interaction with temperature.

**Figure 3:**
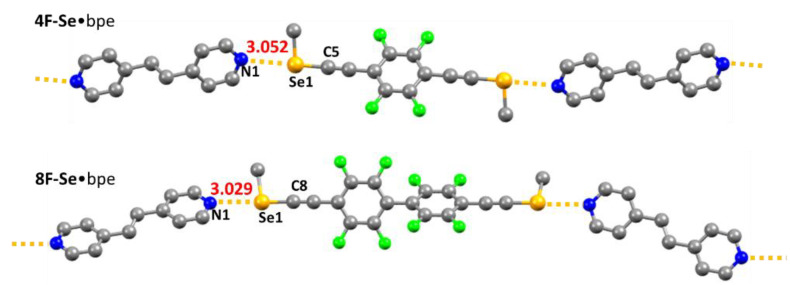
Details of the solid-state structures of 1:1 co-crystals of **bpe** with **4F-Se** and **8F-Se** at RT. Hydrogens atoms are omitted for clarity.

**4F-Se•bpe** crystallizes in the monoclinic system, SG P2_1_/n, with both molecules located on inversion centers, while **8F-Se•bpe** crystallizes in the monoclinic system, SG C2/c, with the **bpe** molecule located on an inversion center and the **8F-Se** Ch donor on a 2-fold axis. The torsion angle between the two aromatic rings in **8F-Se** amounts to 57.5(1)° at RT and to 55.4(1)° at 150 K, i.e., very close to that found in the crystal structure of **8F-Se**. Both co-crystallizations with **bpe** resulted in the formation of 1:1 cocrystals **4F-Se**•**bpe** and **8F-Se**•**bpe**. In both systems, the 1D chains assembled by ChB motifs develop through short Se•••N contacts (Table 1) with intermolecular distances at RT of 3.052(2) Å in **4F-Se**•**bpe** and a shorter 3.029(4) Å distance in case of **8F-Se**•**bpe,** suggesting a stronger activation of the σ-hole in Se-atoms in the latter (Figure 3). This slight strengthening with the **8F-Se** ChB donor is enhanced at low temperatures (150 K) with the Se•••N distances decreasing to 3.005(2) and 2.958(2) Å in **4F-Se•bpe** and **8F-Se•bpe**, respectively.

The strength of the Se•••N interactions in these 1D motifs is also manifested by their almost linear directionality, as the (C≡)C−Se•••N angles are very close to 180° with **4F-Se**, and even closer with **8F-Se** (Table 1).

In conclusion, it appears that replacement of the p-tetrafluorophenylene core in **4F-Se** by the extended p-octafluorobiphenylene one in **8F-Se** not only preserves the robustness of the 1D chalcogen-bonded motif in cocrystals with ditopic Lewis bases such as bpe, but also strengthens it, albeit to a limited extend. This strengthening provides, however, an incentive to prepare even longer ChB donors based on these (chalcogenoalkyl)alkynyl derivatives, toward the elaboration of more complex, eventually porous, systems stabilized by such non-bonding σ-hole-based intermolecular interactions.

## 3. Materials and Methods

General Information. Oxygen- and moisture-sensitive experiments were carried out under a dry oxygen-free nitrogen atmosphere using standard Schlenk techniques. THF was dried using a commercial solvent purification system from Inert Technology. The NMR spectra were recorded on Bruker spectrometers (300 MHz, Bruker, Mannheim, Germany) referenced to residual solvent signals as internal standards. Elemental analyses were performed at BioCIS (Elementar Vario/Perkin Elmer 2400 series, PerkinElmer, Watham, MA, USA). Commercially available compounds 4,4′-dibromoperfluorobiphenyl, 1,2-bis(4-pyridyl)ethane (bpe) and anhydrous triethylamine were purchased and used as received.

*4,4′-Bis(trimethylsilylethynyl)-perfluorobiphenylene* **2**. 4,4′-dibromoperfluorobephenyl (1.5 g, 3.3 mmol) was placed in an oven-dried 100 mL round bottom flask. Anhydrous trimethylamine (50 mL) was added under argon followed by TMS-acetylene (1.4 mL, 9.86 mmol, 3 eq). PdCl_2_(PPh_3_) (230 mg, 0.32 mmol, 0.1 eq.) and CuI (62 mg, 0.32 mmol, 0.1 eq.) were then added to the reaction mixture under argon and the mixture was refluxed overnight. The reaction mixture was cooled down to room temperature and the precipitate formed was filtered off. Trimethylamine was evaporated using rotary evaporator under reduced pressure and the crude solid residue was subjected to flash column chromatography on silica gel for purification (eluent: petroleum ether) to afford **2** (1.1 g, 70%) as a white solid. *R_f_* = 0.5 (petroleum ether); ^1^H NMR (300 MHz, CDCl_3_): δ 0.33 (s, 18H); ^19^F NMR (300 MHz, CDCl_3_): δ −135.5, −138.5.

*4,4′-Diethynylperfluorobiphenylene* **3**. Compound **2** (150 mg, 0.30 mmol) was dissolved in tert-butanol (20 mL) under argon, and potassium carbonate (400 mg, 2.9 mol, 9.6 eq.) was added at 30°C. Reaction was monitored by TLC and 0.1 mL of MeOH was added in an interval of one hour until all the starting material was consumed. Caution: Use of methanol in excess results in the formation of the methoxyacetylene derivative. Reaction mixture was filtered over Celite^®^ and the solution was evaporated using rotary evaporator under reduced pressure. The residue was subjected to flash column chromatography on silica gel (eluent: petroleum ether) for purification to afford **3** (94 mg, 89%) as white solid. *R_f_* = 0.4 (petroleum ether); ^1^H NMR (300 MHz, CDCl_3_): δ 3.78 (s, 2H); ^19^F NMR (300 MHz, CDCl_3_): δ −135.1, −137.8.

*4,4′-Bis(selenomethylethynyl)-perfluorobiphenylene* **8F-Se**. Compound **3** (240 mg, 0.69 mmol) was placed in an oven-dried 100 mL round bottom flask under inert atmosphere. Dry THF (20 mL) was added under argon flow and the solution was cooled to −78 °C. A solution of nBuLi (2.5 M in hexane, 0.624 mL, 1.52 mmol, 2.2 eq.) was added and the reaction mixture was stirred for half an hour. A finely ground dry Se powder (120 mg, 1.52 mmol, 2.2 eq.) was added under argon flow at −78 °C and the reaction mixture was gradually allowed to warm to RT. After 5 h, MeI (0.094 mL, 1.52 mmol, 2.2 eq.) was added dropwise to the dark red solution and stirring was continued overnight. Reaction was quenched with saturated aqueous ammonium chloride solution (5 mL for 1 mmol of alkyne). The mixture was extracted with diethyl ether (2 × 30 mL). The combined organic layers were dried over Na_2_SO_4_, filtered and concentrated under reduced pressure. The obtained crude product was subjected to column chromatography (eluent: petroleum-ether) to afford **8F-Se** (120 mg, 52%) as light-yellow solid. *R_f_* = 0.3 (petroleum ether). Mp: 142–144 °C; ^1^H NMR (300 MHz, CDCl_3_): δ 2.49 (s, 6H); ^19^F NMR (300 MHz, CDCl_3_): δ −136.6, −138.5. ^13^C NMR (300 MHz, CDCl_3_): δ 10.2, 83.2, 89.2, 106.9, 142.2, 145.4, 148.6. Anal. Calcd. for C_18_H_6_F_8_Se_2_: C, 40.62; H, 1.14; found: C, 41.73; H, 1.31. The slightly too large C content can be attributed to some partial decomposition.

*4,4′-Bis(selenomethylethynyl)-perfluorobenzene* **4F-Se**. This was synthesized following the same strategy starting with 1,4-diiododtetrafluorobenzene, as also described in the previous report [26].

Co-crystal Preparation

***4F-Se****•**bpe***. To a solution of **4F-Se** (7 mg) in EtOAc (0.5 mL) was layered with 1,2-bis(4-pyridyl)ethane (3.3 mg, 1 equiv.) dissolved in EtOAc (0.5 mL). Slow evaporation of solvent resulted in the formation of yellow plate-shaped crystals. Mp: 103 °C; Anal. Calcd. for C_24_H_18_F_4_N_2_Se_2_: C, 50.72; H, 3.19; N, 4.93; found: C, 49.40; H, 3.49; N, 4.47.

***8F-Se****•**bpe*****.** To a solution of **8F-Se** (9 mg) in EtOAc (0.5 mL) was layered with 1,2-bis(4-pyridyl)ethane (3.1 mg, 1 equiv.) dissolved in EtOAc (0.5 mL). An overnight slow evaporation of solvent resulted in the formation of yellow plate-shaped crystals. Mp: 125–127 °C. Anal. Calcd. for C_30_H_18_F_8_N_2_Se_2_: C, 50.29; H, 2.53; N, 3.91; found: C, 50.52; H, 2.62; N, 3.86.

Crystallography

Data collections at RT were performed on an APEXII Bruker-AXS diffractometer (Mannheim, Germany) equipped with a CCD camera and data collections at 150 K on a D8 VENTURE Bruker AXS diffractometer. Structures were solved by direct methods using the SIR97 program and then refined with full-matrix least-square methods based on F^2^ (SHELXL-97) [31] with the aid of the WINGX program [32]. All non-H atoms of the molecules were refined anisotropically, and hydrogen atoms were introduced at calculated positions (riding model), included in the structure factor calculations but not refined. Details about data collection and solution refinement are given in Table 2. CCDC No. 2090416-2090420. contains the supplementary crystallographic data for this paper. These data can be obtained free of charge via http://www.ccdc.cam.ac.uk/conts/retrieving.html.

Theoretical Calculations

Molecular structures of **4F-Se** and **8F-Se** have been optimized in gas phase (vacuum) with Gaussian 09 software using density functional theory [33]. B3LYP functional was used, completed with D3 dispersion Grimme dispersion correction [34]. The def2-TZVPP basis set was employed for all atoms. Frequency calculations were performed in order to check that true energy minima were obtained. Isosurfaces of electron density (ρ = 0.002 a.u.) mapped with the corresponding total electrostatic potential were calculated and drawn with AIMAll software [35].

## Figures and Tables

**Table 1:** Structural characteristics of the ChB interactions in co-crystals.

Co-Crystal	T (K)	Se•••N dist. (Å)	RR	C−(Se)•••N Angle (°)
**4F-Se•**bpe	296	3.052 (2)	0.88	172.1 (1)
**8F-Se•**bpe	296	3.029 (4)	0.87	176.7 (1)
4**F-Se•**bpe	150	3.005 (2)	0.87	172.7 (1)
**8F-Se•**bpe	150	2.958 (2)	0.85	177.3 (1)

**Table 2:** Summary of crystal data.

Title 1	8F-Se	4F-Se•bpe (RT)	4F-Se.bpe (150 K)	8F-Se•bpe (RT)	8F-Se•bpe (150 K)
CCDC number	2090416	2090417	2090418	2090419	2090420
Formula	C_18_H_6_F_8_Se_2_	C_24_H_18_F_4_N_2_Se_2_	C_24_H_18_F_4_N_2_Se_2_	C_30_H_18_F_8_N_2_Se_2_	C_30_H_18_F_8_N_2_Se_2_
FW	532.15	568.32	568.32	716.38	716.38
Crystal system	triclinic	monoclinic	monoclinic	monoclinic	monoclinic
Space group	$P\bar{1}$	P2_1_/n	P2_1_/n	C2/c	C2/c
*a*/Å	8.4551 (6)	12.6631 (8)	12.6624 (16)	22.529 (2)	21.575 (2)
*b*/Å	8.6842 (5)	5.7143 (4)	5.6541 (8)	6.2237 (6)	6.1938 (5)
*c*/Å	12.4001 (9)	16.6845 (13)	16.341 (2)	21.539 (3)	21.412 (2)
α/°	91.956 (2)	90.00	90.00	90.00	90.00
β /°	108.704 (2)	105.503 (4)	105.367 (4)	105.862 (7)	105.762 (3)
γ /°	94.837 (2)	90.00	90.00	90.00	90.00
*V*/Å^3^	857.52 (10)	1163.38 (14)	1128.1 (3)	2905.1 (6)	2753.7 (4)
*Z*	2	2	2	4	4
*D_c_*/g cm^−3^	2.061	1.622	1.673	1.638	1.728
*T/*K	150 (2)	296 (2)	150 (2)	296 (2)	150 (2)
μ / mm^−1^	4.395	3.224	3.325	2.620	2.764
*F*(000)	508	560	560	1408	1408
Refl. collected	40049	7490	7242	13879	18466
Refl. indep. (R_int_)	3944 (0.0583)	2635 (0.0489)	2570 (0.0409)	3295 (0.0439)	3152 (0.0426)
Refl. Obs. [I > 2σ(I)]	3449	1779	1985	2005	2568
GOF on *F*^2^	1.076	1.018	1.046	1.014	1.126
*R_1_* [I > 2σ(I)] (all)	0.0264 (0.0332)	0.0453 (0.080)	0.0373 (0.0559)	0.0419 (0.0842)	0.0333 (0.0452)
*wR_2_* [I > 2σ(I)] (all)	0.0608 (0.0651)	0.0966 (0.107)	0.0795 (0.0853)	0.1031 (0.1224)	0.0680 (0.0725)
∆ρ_max,min_/e Å^−3^	+0.508, −0.933	+0.899, −0.606	+0.561, −0.514	0.546, −0.297	+0.573, −0.461

## Data Availability

Data is contained within the article.

## Sample Availability

Samples of the compounds are available from the authors upon request.
